# Ca^2+^-Regulated Photoproteins: Effective Immunoassay Reporters

**DOI:** 10.3390/s101211287

**Published:** 2010-12-10

**Authors:** Ludmila A. Frank

**Affiliations:** Institute of Biophysics, Siberian Branch of the Russian Academy of Sciences, Akademgorodok, 50, Krasnoyarsk, 660036, Russia; E-Mail: lfrank@yandex.ru; Tel.: +8-391-249-44-30; Fax: +8-391-243-34-00

**Keywords:** bioluminescence, Ca^2+^-regulated photoprotein, immunoassay, PCR-ELISA, multiplex assay, re-engineered photoproteins

## Abstract

Ca^2+^-regulated photoproteins of luminous marine coelenterates are of interest and a challenge for researchers as a unique bioluminescent system and as a promising analytical instrument for both *in vivo* and *in vitro* applications. The proteins are comprehensively studied as to biochemical properties, tertiary structures, bioluminescence mechanism, *etc*. This knowledge, along with available recombinant proteins serves the basis for development of unique bioluminescent detection systems that are “self-contained”, triggerable, fast, highly sensitive, and non-hazardous. In the paper, we focus on the use of photoproteins as reporters in binding assays based on immunological recognition element—bioluminescent immunoassay and hybridization immunoassay, their advantages and prospects.

## 1. Introduction

**Figure 1.**
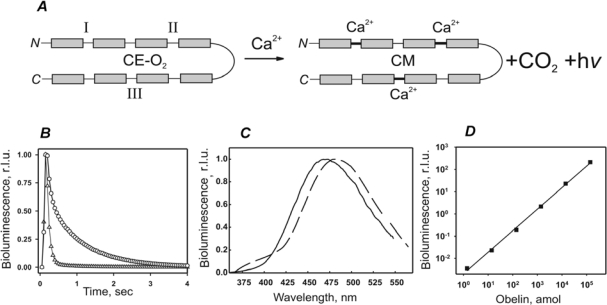
Bioluminescence of Ca^2+^-regulated photoprotein. **(A)** Scheme for the reaction: photoprotein is a complex of single-chain polypeptide, containing Ca^2+^-binding sites (I, II, III) and pre-oxidized coelenterazine (CE-O_2_). The binding of Ca^2+^ results in coelenterazine decarboxylation yielding a stable complex of polypeptide, three Ca^2+^ and coelenteramide (CM), carbon dioxide and a quantum of light. **(B)** Kinetics of aequorin (-○-) and obelin (-Δ-) bioluminescence signals. **(C)** Bioluminescence spectra of aequorin (solid line) and obelin (dashed line). **(D)** Obelin amount versus bioluminescence.

Ca^2+^-regulated photoproteins of luminous marine coelenterates [1] are stable complexes of apophotoprotein (a single-chain polypeptide with molecular mass around 20 kDa) and a pre-oxidized substrate molecule—peroxycoelenterazine—which is strongly but non-covalently immobilized in the protein hydrophobic cavity [2,3]. The primary structures of photoproteins known today reveal high homology and all have three Ca^2+^-binding sites [4]. Upon Ca^2+^ addition, proteins undergo conformational changes that cause substrate decarboxylation resulting in the protein bound product—coelenteramide—in an excited state (Figure 1A). The energy of the reaction releases as a flash of blue light, centered at 469–495 nm, depending on the protein origin (Figure 1C). Excess of calcium ions induces a rapid reaction, with a decay period of activated intermediate less than 1 sec (Figure 1B). Bioluminescence (BL) is detected in a luminometer allowing injection of calcium solution and simultaneous measurement of light. The BL produced by photoproteins overlaps several logs of concentration with a linear relationship between protein amount (Figure 1D) and BL and high signal-to-background ratio. In contrast to the luciferase reaction, photoprotein bioluminescence does not depend on oxygen or substrate concentration. A high quantum yield of the reaction, a virtual absence of the background BL signal, and a high sensitivity of modern photometers make the photoprotein detection down to attomole level possible.

To date, Ca^2+^-regulated photoproteins have been discovered in more than 25 different coelenterates [1]. Among them, the best investigated are aequorin, named for its origin in the jellyfish *Aequorea* and obelin, from the hydroid polyp *Obelia*. The cDNAs of the proteins were cloned and expressed in a high yield in bacterial cells. Recombinant apoproteins are effectively activated with a synthetic coelenterazine under calcium-free conditions in the presence of O_2_ and reducing reagent [5–8]. The proteins are comprehensively studied as to biochemical and biophysical properties, tertiary structures, bioluminescence mechanism *etc*. This knowledge along with practically unlimited amounts of recombinant proteins serves the basis for development of unique bioluminescent detection system that is “self-contained”, triggerable, fast, highly sensitive, and non-hazardous.

One of the applications of photoproteins is conditioned by their sensitivity to calcium ions in a physiological range of concentration where those are used as a valuable probe for studying intracellular Ca^2+^ [9,10]. But of no less interest is the application of photoproteins (at saturating Ca^2+^ concentrations) as effective reporters in binding assay of various biologically active substances. Here we focus on the use of aequorin and obelin in: (a) bioluminescent immunoassay, (b) bioluminescent PCR-ELISA, (c) assay in tandem with the other reporters, and on analytical application of re-engineered photoproteins as well. The data over last 15 years will be reviewed.

## 2. Bioluminescent Immunoassay

Publications have described the use of photoproteins as immunoassay reporters since the early 1990s when the active recombinant proteins became available. In estimating photoproteins as labels, several obvious advantages of those were taken into account: practically unlimited linear range of bioluminescence, the lack of background, the simplicity the reaction is triggered with, the availability of modern registration devices and the absence of toxicity. The application of photoproteins as tracers in binding assays required that their tolerance to chemical modifications be ascertained—conjugation with the other molecules (antigens or antibodies) or binding of definite anchor groups serving as effective bridges between the molecules of the immune complex. Erikaku *et al*. [11] chemically conjugated apoaequorin with human necrosis factor-α (hTNF) antibodies (Fab fragment). They found that aequorin activity in the conjugate was regenerated (by incubation with coelenterazine) up to only 10% of the free photoprotein activity. Nevertheless, the sensitivity of the hTNF assay (standard samples) was one attomole, approximately 100-fold higher than that achievable using the analogous conjugate with β-galactosidase. Later it was demonstrated that photoproteins (preliminary coelenterazine-activated apophotoproteins) are essentially more stable to chemical modifications than apoproteins and their active conjugates can be easily obtained applying commercially available reagents and cross-linkers [12–16]. The conjugates in solution as well as in frozen and lyophilized states were found to be stable and easily stored. Consequently, in the following years a great number of publications described photoprotein-based immunoassays of different analytes of clinical interest – hormones, interleukins, oncomarkers, *etc*. [12–26], and infections [26–28]. The assays were carried out in different formats (sandwich, competitive), and performed in solid-phase, homogeneous and even flow injection [29] variants. The papers considered investigations of model samples as well as sera, saliva, mucous of patients and experimental animals.

Of special interest are the evaluations of the aequorin-based thyrotropin (TSH) assay undertaken at the Pathology and Laboratory Medicine Departments of Emory University and the Veterans Administration Hospital (Atlanta, USA) [20]. The authors measured serum TSH in 153 euthyroid individuals with thyroidal illnesses (primary hypo- and hyperthyroids, thyroids cancer, *etc*.) applying bioluminescent methods (developed and marketed by SeaLite Inc., USA) and compared the results with the data obtained by commercially available Nichols, ACS-180 and TOSOH methods. They found that aequorin-based method has the required performance characteristics (e.g., the functional sensitivity of 0.017 mIU/L, which is higher than in the cases of ACS-180 and TOSOH assays) and is “clearly qualified as a last, third-generation TSH assay”. In [19], reproducibility of bioluminescent immunoassay of TSH and thyroxin in sera of patients, treated by different medicines (more than 20 items) was investigated. No significant influence of substances or their metabolites on the assay results was observed, this pointing to the high reliability of the assay.

**Figure 2.**
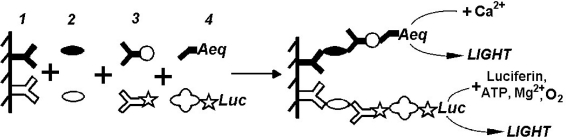
Generalized scheme for dual bioluminescent immunoassay using aequorin in tandem with firefly luciferase, according to [31]. The surface was activated by antibodies of two types (1) against two antigens (2). Secondary antibodies (3) were tagged with digoxigenin and biotin. The sandwiches, formed on the surface were detected using conjugate aequorin-antidigoxigenin Fab fragment and biotinylated luciferase-streptavidin complex (4). Ca^2+^ injection triggered flash-type aequorin bioluminescence and then the mixture of luciferin, ATP and Mg^2+^ was placed into the wells to initiate luciferase bioluminescence.

The unique bioluminescence nature of photoproteins (triggering with Ca^2+^, flash-type signal) conditioned their application in tandem with other reporters to provide simultaneous detection of several antigens in one sample. The tandem luminescence concept was demonstrated by Adamczyk *et al*. [30] who applied aequorin and acridinium-9-carboxamide labels in a model assay system. The signals were triggered sequentially by injection of Ca^2+^ solution followed by basic peroxide, with a total read time of 20 s. It was concluded that the relatively short assay time and the well-resolved signals made this format suitable for the development of dual analyte high-throughput assays. Ito and co-authors [31] developed highly sensitive and rapid immunoassay of two antigens involving aequorin and firefly luciferase labels. The authors detected two couples of antigens—prostatic acid phosphatase (PAP) with prostate specific antigen (PSA) (measurable ranges of 0.04–100 and 0.2–200 ng/mL, respectively) and PSA with alphafetoprotein (AFP) (measurable ranges of 0.2–200 and 1.95–1000 ng/mL, respectively). Figure 2 illustrates the proposed tandem immunoassay. With the developed technique applied, PAP, PSA and AFP were detected in standard and then in clinical sera. The values obtained from the developed simultaneous assay were in good correlation with those derived from conventional methods. Worthy of notice is the approach further developed for multiplex hybridization assay with aequorin used in tandem with several reporters (see e.g., [50]).

The data considered above obviously testify to the high level of research on photoprotein-based immunoassay. Actually, the state-of-the-art in the field allows wide-scale introducing bioluminescent analytical technologies into practical use under clinical conditions.

## 3. Bioluminescent Immunoassay for PCR Products Quantitation

The polymerase chain reaction (PCR) has proved to be of great value in diagnostic research. Its ability to amplify specific nucleic acid sequences several million-fold as much has facilitated detection of a small number of DNA copies. For some diagnosis it is necessary to establish the presence or absence of the sequence of interest (diseases associated with infectious agents, gene deletions, SNP, *etc*.). In some cases quantitative PCR analysis is important in order to evaluate therapy effectiveness, detect gene expression through reverse transcriptase-polymerase chain reaction (RT-PCR), *etc*. The traditional approach of PCR products calculation was (and is sometimes!) their visualization after separation on agarose gel by DNA bands density, or scintillation counting of radiolabeled products. The obvious shortcomings of these techniques—low sensitivity, bulkiness to perform or the need to use radioactive materials—have hampered implementation of these assays in routine settings. That is why new non-radioisotope methods allowing quantification of PCR products are being intensively developed.

In this respect some features are to be taken into account. Firstly, determination of the original number of copies requires a constant relationship between the product yield and the input molecules. But as the number of cycle increases the amplification process diverges from an exponential product generation and reaches a plateau, so quantitative analysis must be restricted to the exponential phase of the reaction. At the same time, the smaller number of cycles results in decrease of the product amount to be analyzed, so the detection sensitivity should be extremely high. Secondly, despite the use of high temperatures, non-specific primer annealing may occur during PCR, and errors are exponentially amplified through multiple cycles. In order to discriminate these products and to obtain a higher level of specificity, a hybridization step must be included into assays. And thirdly, attention should be paid to the dynamic range of instruments used for quantitative measurements.

**Figure 3.**
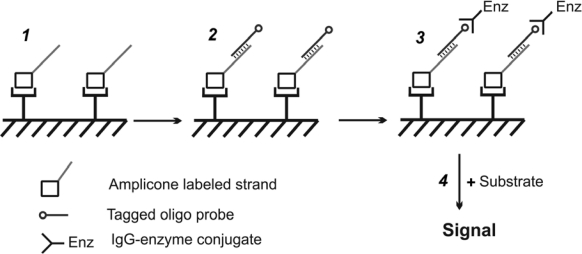
Scheme for PCR-ELISA method developed by Vlieger *et al*. [30]. **1**. amplicon labeled strands immobilization on the surface; **2.** hybridization with tagged probe; **3.** immuno-enzymatic complex formation; **4.** amplicon detection through enzyme reaction.

One of the approaches meeting all the demands is the PCR-ELISA method, proposed by Vlieger *et al*. in 1992 [32]. The merit of the method lies in the combination of PCR, oligonucleotide-hybridization and enzyme immunoassay capabilities. In general the assay includes three steps (Figure 3): PCR is carried out using specifically-labeled forward primer and amplicons (labeled strand) are immobilized on a surface (covalently or by affinity coupling); hybridization with tagged probe; immuno-enzymatic “recognition” and evaluation of probe-DNA duplexes. In some cases tagged probe is added to the mixture after amplification and hybridization is performed using temperature denaturation of amplicons. Then hybrids carrying two different labels are immobilized on the surface. The same technique is applicable to analyze small amounts of mRNA via reverse transcriptase-polymerase chain reaction (RT-PCR) followed by ELISA [33]. Since the early 90ies several modifications of the method have been developed, based on colorimetric, fluorescent and chemiluminescent detection [34,35], mostly in microplate format.

In 1996 Siddiqi *et al*. proposed the use of the photoprotein aequorin as a bioluminescent reporter [36]. The aequorin-based assay of IL-2 PCR product was compared to electrochemiluminescence-based (ECL) assay. It was found that BL assay detected down to 1 × 10^13^ copies (14 cycles), while ELC—1.5 × 10^14^ copies (18 cycles). The detection rate for ECL is 48 samples/h, and for aequorin-based microplate assay—one sample/3 second, so the BL assay turned out to be more rapid, sensitive and inexpensive than ECL.

In the same year Xiao *et al*. [37] applied an aequorin-based immunoassay for quantitation of RT-PCR amplified cytokine mRNA. The cDNA was synthesized from total mRNA isolated from stimulated murine splenocytes, and amplified for IL-2, IL-4, IL-10 and IFN-γ. The technique enabled detection of as low as 40 amol of amplified cytokine products or 500 copies of templates using 28 cycles. This detection level is essentially lower than that of the colorimetry-based enzymatic immunoassay level (fmol). Besides, the BL-based assay has an essentially higher dynamic range and smaller coefficient of variation, especially for small amounts of product (less than 5% *versus* 20%). Similar investigations were carried out by Actor *et al*. [38]. They detected RT-PCR amplified mRNA for 10 cytokines in stimulated and unstimulated human cells using aequorin-based technology. The significant advantages of bioluminescent method as opposed to the radioactive one were revealed, *i.e.*, higher sensitivity and ability to analyze products in an automated microtiter plate format. Shortly after that, bioluminescent immunoassay in combination with internal standard and competitive coamplification technique [39] was demonstrated as an accurate tool for DNA and mRNA quantitation in many medico-biological applications: to quantify human immunodeficiency virus (HIV-1) DNA or RNA in plasma, vaginal lavage and cultured cells [40]; to evaluate efficacy of conventional and DNA vaccinations against *Helicobacter pylory* [41]; and to investigate gene expression under infectious disease conditions [42–46].

Within the framework of the review we focus only on the methods using immunological recognition elements but not less interesting and prospective are the techniques involving aequorin-biotin (or -streptavidin) or aequorin-oligonucleotide conjugates [47–50].

## 4. Analytical Application of Re-Engineered Photoproteins

Re-engineered (genetically modified) photoproteins have been widely described in literature. Part of them were obtained in the course of fundamental studies on elucidation of the role of some amino acids in bioluminescent reactions, other ones—in applied studies aimed at the creation of photo-proteins with novel useful properties (altered bioluminescence spectrum and kinetics, Ca^2+^-sensitivity, *etc*.). Of course, this boundary is far from being strict and in reality fundamental and applied investigations interact and are complementary.

Attempts to vary the bioluminescence of photoproteins (to alter their spectral characteristics, kinetics, Ca^2+^-sensitivity, *etc*.) using site-directed mutagenesis have been undertaken since their cDNAs were cloned. The W86→F86 substitution in the aequorin molecule, for instance, was found to give an additional violet band in the bioluminescence spectrum, with a violet-to-blue ratio of 0.4 [51]. The corresponding W92→F92 substitution in the obelin molecule gives the same effect with a violet-to-blue ratio of 1 [52]. This spectrum changes take place because the reaction product—excited state coelenteramide—can form several kinds of light emitters, depending on the surroundings [53]. Thus, the site-directed replacement of amino acids in coelenterazine-binding pocket that changes its polarity and hydrogen-bond network can result in the formation of different emitters.

**Figure 4.**
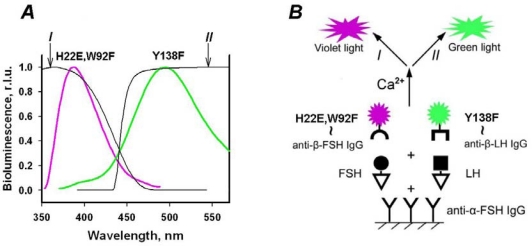
Color photoprotein obelin mutants as reporters in dual immunoassay according to [56]. **(A)** Bioluminescence spectrum of “violet” (W92F, H22E) and “greenish” (Y138F) obelin mutants (thick lines); band-pass optical filters *I* and *II* transmission (thin lines). **(B)** Simultaneous dual-color bioluminescence immunoassay of LH and FSH: the surface was activated with anti-α-FSH (the same for both hormones) IgG. Sandwich immunocomplex formed at incubation of hormones and conjugates of obelin mutants with corresponding hormones anti-β-subunits (specific for every hormone) IgGs mixture. Reporters’ bioluminescence was simultaneously triggered by single injection of Ca^2+^ solution, divided using band-pass optical filters and measured with a two-channel photometer.

Rationally directed re-engineering of photoproteins came into being in 2000, when the spatial structures of aequorin and obelin were solved [2,3] and as a consequence, the amino acids essential for the bioluminescence reaction were proposed. In the case of obelin it was found that mutagenesis of W92, W179, H22, H175, Y138, and Y190 residues changes the bioluminescence activity and (or) spectrum [54–56]. Some of the mutants had high bioluminescence activity and stability, thus allowing their use as reporters. The “violet” W92F, H22E and “greenish” Y138F obelins were applied in a one-stage dual-color simultaneous immunoassay of two gonadotropic hormones—follicle-stimulating hormone (FSH) and luteinizing hormone (LH) in one serum sample [56]. Bioluminescence of the reporters was simultaneously triggered by a single injection of Ca^2+^ solution, divided using band-pass optical filters and measured with a two-channel photometer (Figure 4). The sensitivity of the simultaneous bioluminescence assay was close to that of a separate radioimmunoassay.

To design aequorin with unique bioluminescent properties two strategies were applied: site-directed mutagenesis and incorporation of coelenterazine analogues that retained bioluminescence activity with a concomitant spectrum shift [57,58]. In a recent publication, the use of coelenterazine analogues was combined with site-directed incorporation of non-natural amino acids to modulate spectrum emission [59]. It should be noted however that all these aequorins with altered bioluminescence displayed shifted, but rather wide signals, with essential overlapping areas, that makes signal discrimination rather difficult. Simultaneous, dual-analyte immunoassay for 6-keto-prostaglandin-FI-alpha and angiotensin II, two important cardiovascular molecules, was successfully realized in [60]. In this particular case the signals of both aequorin variants were triggered by Ca^2+^ and resolved from one another using the differences in decay kinetics.

Multi-analyte approach allows intensification of the assay procedure especially in the case when, for proper diagnostics, it is recommended to detect several analytes in one sample. Simultaneous determination may be useful for large-scale investigation and may offer advantages in terms of cost and labor savings.

One more type of re-engineered variants is a photoprotein genetically fused with the proteins of interest. The gene-fusing technique has become an increasingly useful tool in biomedical research. For instance, the construction of bifunctional molecules containing bio-specific and reporter modules provides effective markers to be applied in both *in vivo* and *in vitro* assays. The main advantages of the labels obtained as fused proteins are: (1) the lack of bulky chemical crosslinking stages, (2) high activity of enzyme module, not subjected to any chemical treatments and (3) usually simple way to purify chimeric proteins with affinity chromatography.

The Ca^2+^-regulated photoproteins hold much promise as reporter modules providing high sensitivity to the assays under development. It is not surprising that one of the first bioluminescent immunoassays was proposed on the basis of aequorin genetically fused with immunoglobulin heavy-chain [61]. Antibody-aequorin chimera was expressed in mammalian cells and possessed photoprotein bioluminescent activity, close to that of WT aequorin and high antibody binding ability and was used as an immunoassay label. Photoproteins fused with *Staphylococcus aureus* protein A or its immunoglobulin-binding fragment Z are universal labels for IgG detection [62,63]. Photoproteins fused with biotin-acceptor peptide, are biotinylated post-translationally and can be utilized as reporter molecules in assays based on avidin-biotin technology [64,65]. It was established that aequorin being fused with biologically active polypeptides, “works” successfully as an *N*-terminal or *C*-terminal partner [66,67]. Thus the photoprotein module has proved to be a sensitive reporter applicable for obtaining fusion proteins and the further development of such constructions depends on availability of corresponding selective recognition elements.

## 5. Conclusions

The Ca^2+^-regulated photoproteins of luminous marine coelenterates were discovered at the beginning of the 60ies and have been of interest and challenge for researchers as a unique bioluminescent system and as a promising analytical instrument ever since. The availability of recombinant proteins has stimulated the studies on their applications as supersensitive tracers in bioluminescent binding assays. Convincingly good experimental results, commercial availability of photoproteins and their conjugates as well as of various photometers of high sensitivity allow wide-scale introduction of bioluminescent analytical technologies into practical use in medicine and biotechnology. This review is concerned only with immunoassay technologies, but the spectrum of applications of photoproteins as supersensitive analytical tools is essentially broader and the number of papers on the topic is enormous. More information can be found elsewhere [68–70].

No doubt, bioluminescence assays based on Ca^2+^-regulated photoproteins look highly promising from the standpoint of increasing demands of modern science. The main directions here are the creation of multianalytical systems, assay miniaturization, and the development of high-throughput technologies and sensors.
